# Iatrogenic Horner syndrome following internal jugular vein catheterization in an infant: a case report and literature review

**DOI:** 10.1097/RC9.0000000000000875

**Published:** 2026-08-26

**Authors:** Hela Oueslati, Asma Jabloun, Fatma Trabelsi, Imen Kalfat, Souha Laarif, Youssef Hellal

**Affiliations:** a Faculty of Medicine of Tunis, University Tunis El Manar, Tunis, Tunisia; b Department of Pediatric Surgery, Habib Thamer Hospital, Tunis, Tunisia

**Keywords:** case report, catheterization, Horner syndrome, internal jugular vein, sympathetic nervous system

## Abstract

**Introduction:**

Horner syndrome (HS) is a rare but recognized complication of internal jugular vein (IJV) catheterization in children, resulting from injury to the cervical sympathetic pathway. Neonates and infants are particularly vulnerable due to the compact anatomy of the neck.

**Presentation of the case:**

We report a 38-day-old female infant who developed ipsilateral HS following open surgical placement of a Broviac catheter via the right IJV. The catheter was inserted under local anesthesia with sedation for prolonged intravenous therapy. Within 24 hours postoperatively, the patient presented with right-sided ptosis, miosis, and apparent enophthalmos, without any other neurological deficits. The diagnosis of iatrogenic HS was made based on the typical clinical features and the close temporal relationship with the procedure. Management consisted of close observation with serial neurological and ophthalmological examinations, and the catheter was left in place. Progressive improvement was noted within 48 hours, with complete resolution of symptoms by 1 week.

**Discussion:**

HS following IJV catheterization is uncommon in pediatric patients, with only a few cases reported in the literature. Although most cases occur after percutaneous cannulation, this complication may also arise after open surgical approaches, even under direct visualization. Proposed mechanisms include direct injury to the sympathetic chain, compression from a hematoma or edema, and ischemic neuropraxia. The prognosis is generally favorable, with spontaneous recovery in most cases.

**Conclusion:**

This case highlights that iatrogenic HS can occur after open surgical IJV catheterization in infants. Awareness of this rare complication, careful surgical technique, and prompt recognition are essential to ensure optimal outcomes.

## Introduction

Horner syndrome (HS), also known as Claude Bernard–Horner syndrome, results from disruption of the oculosympathetic pathway. It is characterized by ipsilateral ptosis, miosis, and apparent enophthalmos, occasionally accompanied by anhidrosis^[^1,2^]^. In pediatric practice, HS may arise following neck or thoracic interventions, including central venous catheterization via the internal jugular vein (IJV). Although rare, both percutaneous cannulation and surgical exposure of the IJV have been implicated^[^3^]^. The present report highlights the occurrence of transient HS following open surgical Broviac placement through the IJV in a premature infant, emphasizing that this complication may occur even under direct surgical visualization.

HIGHLIGHTS

- Horner syndrome is a rare complication of pediatric internal jugular vein catheterization.
- It may occur even after open surgical catheter placement.
- Infants are particularly vulnerable due to the proximity of cervical anatomical structures.
- Serial neuro-ophthalmologic monitoring is usually sufficient, as the resolution is spontaneous.
- Awareness and meticulous technique are essential to prevent this complication.

This case report has been reported in line with the SCARE checklist^[^4^]^.

## Case presentation

A 38-day-old female infant (weight: 2.7 kg) was admitted with severe sepsis secondary to neonatal necrotizing enterocolitis (NEC), requiring prolonged intravenous antibiotics and parenteral nutrition. She was born prematurely at 36 weeks’ gestation with a birth weight of 2.5 kg and required neonatal intensive care unit hospitalization. After removal of an umbilical catheter and, subsequently, a Jonathan catheter, worsening peripheral venous access combined with the need for long-term parenteral nutrition and antibiotics necessitated durable central access via a Broviac catheter. The right internal jugular route was chosen over the femoral or subclavian veins to reduce the infectious and thrombotic risks of femoral lines in an infant recovering from intestinal inflammation and to avoid the pneumothorax risk of subclavian access in a premature infant. No pre-existing cervical or vascular abnormalities were present prior to the procedure, and the patient was hemodynamically stable, without coagulation abnormalities.

A 2.7 Fr Broviac catheter was surgically inserted via the right IJV under local anesthesia with sedation that was administered and monitored by a pediatric anesthetist. Sedation consisted of intravenous midazolam (0.1 mg/kg, 0.27 mg total) and fentanyl (1 mcg/kg, 2.7 mcg total), with continuous cardiorespiratory monitoring (heart rate, pulse oximetry, and non-invasive blood pressure). Local anesthesia was achieved with infiltration of 0.2 mL of 1% lidocaine (2 mg; 0.74 mg/kg).

The infant was placed in the supine position with a roll beneath both scapulae to achieve mild cervical hyperextension. The head was rotated approximately 45° toward the contralateral side. After local infiltration with lidocaine (2 mg), the right IJV was surgically exposed through a cervical incision without ultrasound guidance. Dissection was technically difficult because of the small caliber of the cervical structures. No arterial puncture occurred, and blood loss was minimal. The patient’s exact length at the time of catheterization was, unfortunately, unavailable. The procedure was performed by a consultant pediatric surgeon.

Proximal ligation of the vein was performed while distal venous control was secured around the catheter after venotomy to maintain catheter fixation and venous control while preserving catheter function.

Within 24 hours, the parents noted asymmetry of the eyelids. Examination revealed right-sided ptosis, miosis, anisocoria, and apparent enophthalmos (Fig. 1) without conjunctival injection, ophthalmoplegia, or additional neurological abnormalities. These findings were consistent with HS secondary to recent IJV surgery.

**Figure 1.**
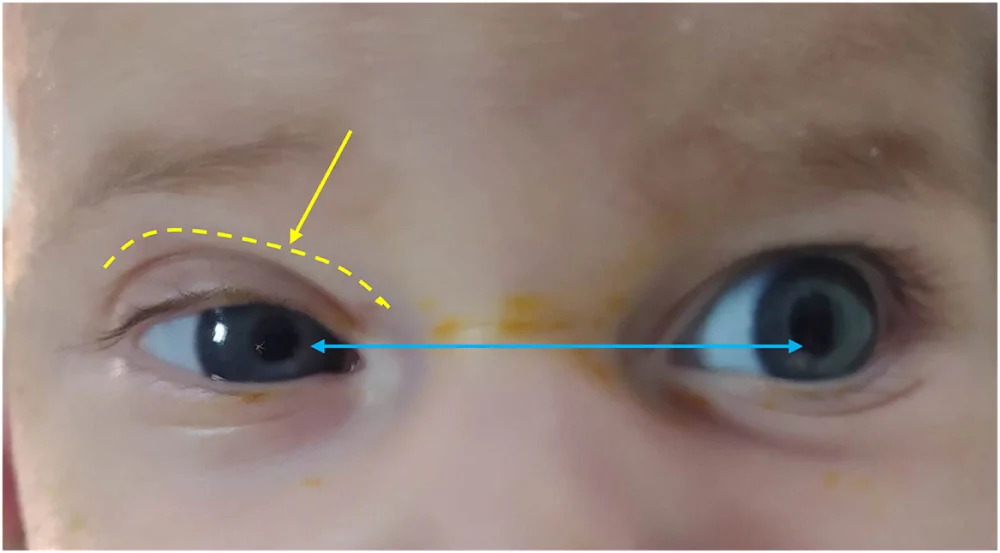
Clinical presentation of the patient, showing right-sided ptosis (yellow arrow and dashed curved line), miosis (blue double-headed arrow), and apparent enophthalmos.

No cervical swelling, hematoma, venous congestion, or focal neurological deficits were observed. Bedside ultrasonography did not demonstrate a hematoma or carotid injury. Ophthalmologic examination confirmed ptosis and anisocoria without additional ocular abnormalities, while neurological evaluation was otherwise unremarkable. Assessment of anhidrosis was difficult in this young infant. Given the stable neurological examination, the absence of compressive signs, and a close temporal relationship to surgery, further neurovascular imaging was deferred.

The catheter functioned well, and management consisted of close clinical observation with serial neurological and ophthalmological examinations. Symptoms improved within 48 hours and resolved completely by postoperative day 7.

## Discussion

The first pediatric case was reported by Parikh in 1972^[^5^]^. Although most cases occur after percutaneous cannulation, surgical placement under direct visualization has also resulted in HS^[^6^]^, underscoring that visualization does not abolish risk. The onset of symptoms varies considerably, ranging from immediately post-procedure to several days later, underscoring that both acute mechanical insult and delayed compressive phenomena may contribute^[^7^]^. The clinical characteristics and outcomes of previously reported pediatric cases are summarized in Table 1^[^5–16^]^.

**Table 1:** Literature review of pediatric Horner syndrome following internal jugular vein catheterization.

Author (year)	Title (journal)	Sex/Age	Medical history/indication for CVC	Catheter technique (percutaneous/surgical)	Imaging guidance (ultrasound)/landmark/not detailed	Symptoms developed	Onset delay	Investigations performed – results	Presumed cause of HS	Management	Resolution & time
Parikh & Parikh (1972)^[^5^]^	Horner’s syndrome: a complication of percutaneous catheterisation of internal jugular vein (Anaesthesia)	F, 3 y	Abdominal trauma → central access	Percutaneous (elective technique)	Landmark (Patient in 20° head-down tilt, head extended and turned left)	Ptosis, miosis, enophthalmos	Early postoperative period	Chest X-ray confirmed catheter position; encephalogram showed no midline shift	Suspected central hypoxia and/or neuropraxia from cannulation maneuvers	Conservative	Partial persistence at discharge; pupil still smaller at 4 months.
Zeligowsky *et al* (1991)^[^7^]^	Horner syndrome: a rare complication of internal jugular vein cannulation (JPEN/J Parenter Enteral Nutr)	M, 18 y	Trauma/multisystem injuries → central access	Percutaneous (multiple attempts)	Landmark	Ptosis, miosis, anhidrosis	9 days	Not detailed	Sympathetic injury (likely neuropraxia or compression)	Conservative	Gradual resolution; complete recovery by 10 weeks.
Silvani *et al* (1993)^[^8^]^	Claude Bernard–Horner syndrome following percutaneous catheterization of the jugular vein in an infant (Cah Anesthesiol)	M, 8 mo	Not detailed	Percutaneous (multiple attempts)	Ultrasound-guided	Ptosis, miosis; laterocervical hematoma noted	Not detailed	Ultrasound: laterocervical hematoma	Compression from laterocervical hematoma	Conservative, management of hematoma	Spontaneous recovery after hematoma resolution.
Williams *et al* (2004)^[^9^]^	Horner’s syndrome following attempted internal jugular venous cannulation	F, 6 y	Admitted for wound debridement under GA; venous access limited due to prior bilateral below-knee and below-elbow amputations from meningococcal septicaemia → central access	Percutaneous (right IJV cannulation not successful → Access gained to the right external jugular vein)	Not detailed	Ptosis, miosis	Immediate postoperative	Ultrasound: no hematoma, no carotid dissection	Direct neuropraxia	Conservative	Improvement by 18 d
Ford & Lauder(2007)^[^10^]^	Case report of Horner’s syndrome complicating IJV cannulation in a child (Pediatr Anesth)	M, 19 mo	Cystic fibrosis; Pseudamonal chest infection → prolonged IV antibiotics → central access	Percutaneous (Seldinger technique) (multiple attempts)	Ultrasound-guided	Ptosis, miosis	Hours after insertion	Chest X-ray confirmed catheter position; ultrasound: no hematoma; neuro exam otherwise normal	Direct neuropraxia	Conservative	Ptosis improved by day 8; anisocoria improved by 5 weeks
Büyüktortop *et al* (2008)^[^11^]^	A case of postganglionic Horner syndrome after catheterization of IJV confirmed with pharmacological tests (Turk J Pediatr)	F, 9 y	High venous ASD, post-closure surgery → central access	Percutaneous	Not detailed	Ptosis, miosis	First postoperative day	Pharmacologic testing (cocaine, phenylephrine, apraclonidine): results consistent with postganglionic lesion; Doppler Ultrasound:normal; CT scans of the chest and neck: normal; Ophtalmological and neurological examinations were unremarkable	Direct postganglionic injury	Conservative (ophthalmological follow-up)	HS persisted at 12 weeks follow-up.
Suominen *et al* (2008)^[^12^]^	Horner’s syndrome secondary to IJV cannulation (J Clin Anesth)	M, 5 y	Ex-preterm, neonatal ileal perforation surgery; emergency laparotomy for obstruction → postop central line for PN + antibiotics.	Percutaneous	Landmark (Rolled towel under shoulders, head rotated 45° left, table 15° head-down; three attempts, carotid puncture occurred)	Ptosis, miosis	72 h	MRI/ MRA: normal	Likely neuropraxia and/or compression after carotid puncture	Conservative	Complete resolution after 5 months
Ahmad & Hayat *et al* (2008)^[^13^]^	Horner′s syndrome following internal jugular vein dialysis catheter insertion (Saudi J Kidney Dis Transplant)	M, 17 y	Chronic renal failure on hemodialysis; required temporary right IJV catheter after failed arteriovenous access.	Percutaneous (multiple attempts)	Not detailed	Ptosis, miosis, swelling and pain in right eye, and decreased visual acuity	Immediate postoperative	Chest X ray: hematoma in the right side of neck with slight tracheal compression; neck & chest CT scan: unremarkable	Sympathetic plexus injury/ hematomata suspected	Conservative	Resolution of anisocoria and ptosis over 4 weeks.
Tobias & Joy (2014)^[^14^]^	Horner syndrome following attempted internal jugular catheter placement in a toddler with congenital heart disease (J Med Cases)	F, 8 mo	ASD, pulmonary stenosis and a small patent ductus arteriosus; Bronchiolitis and respiratory failure → IJV cannulation	Percutaneous (attempted right IJV cannulation, aborted)	Not detailed	Anisocoria, slight ptosis	Immediate postoperative	CT head normal; neck ultrasound: no hematoma;	Direct neuropraxia	Conservative	Slight resolution of ptosis by 3 weeks; anisocoria persisted but improving
Khochtali *et al* (2014)^[^6^]^	Horner’s syndrome following IJV catheterization in an infant (Tunis Med)	Not specified, 2 mo	Post cardiac surgery complicated by sternal osteitis → central access for antibiotics	Surgical open (venotomy with catheter insertion & fixation)	Direct surgical exposure	Ptosis, miosis, pseudo-enophthalmos	Immediate postoperative	Ultrasound: no hematoma or dissection	Direct surgical trauma to cervical sympathetic trunk (mechanical/ischemic from proximal ligation)	Conservative	Progressive spontaneous recovery over ~3 months
Chakraborty *et al* (2021)^[^15^]^	Horner syndrome: an unknown entity after tunneled central venous catheter insertion (J Pediatr Hematol/Oncol)	3 patients, not detailed	Hematopoietic stem cell transplant → tunneled access	Percutaneous	Ultrasound guided	Ptosis, miosis	24 h	Ultrasound: no hematoma	Direct neuropraxia /compression	Conservative	1 patient: resolution in 2 weeks 2 patients: resolution in 2 months
Gomes *et al* (2022)^[^16^]^	Pediatric Horner’s syndrome secondary to IJV cannulation: case report (eOftalmo)	M, 7 mo (premature twin)	Premature twin; Bronchiolitis: → Failed peripheral access for the administration of medications → central access	Percutaneous (multiple attempts)	Not detailed	Ptosis, anhidrosis, miosis	24 h	MRI/CT chest & neck, Doppler IJV: all normal	Traumatic injury to second-order sympathetic neuron	Conservative: Periodic monitoring	After 1 year, no functional visual impairment
Current case	Iatrogenic Horner syndrome following IJV catheterization in an infant	F, 38 days	Severe sepsis → central access for PN and antibiotics	Surgical open Broviac insertion (IJV ligated proximally & distally)	Direct surgical exposure	Ptosis, miosis, enophthalmos	24 h	No imaging (deferred due to clear surgical context and stability)	Mechanical/ischemic injury or compression of sympathetic fibers (likely from proximal ligation/local swelling)	Conservative	Improvement within 48 h; complete resolution by 1 week.

GA, general anesthesia; ASD, atrial septal defect; PN, parenteral nutrition; IJV, internal jugular vein; MRI, magnetic resonance imaging; MRA, magnetic resonance angiography.

Proposed mechanisms include:

- Direct injury to the sympathetic trunk or superior cervical ganglion can occur during puncture or dissection, especially when head rotation exceeds 35–40°, which distorts the normal anatomic relationship between the IJV and the carotid sheath contents^[^6,12^]^
- Compression of sympathetic fibers due to hematoma, vascular ligation, or swelling^[^8,14^]^.
- Anatomical variations, such as overlap between the carotid artery and the IJV, occur in up to 18% of young children^[^17^]^.

In this case, although the exact mechanism cannot be definitively established, transient mechanical or ischemic neurapraxia related to surgical dissection, traction, local edema, or vascular ligation near the cervical sympathetic chain appears most likely.

In retrospect, the degree of cervical rotation used during the procedure may have contributed to the distortion of cervical anatomical relationships. Limiting head rotation to less than 30° when feasible may help reduce tension on adjacent neurovascular structures.

Children are especially vulnerable due to narrower cervical anatomy and fragile neural structures^[^16^]^. Many published cases have followed technically challenging cannulations with multiple puncture attempts^[^10,14^]^, although HS has also occurred after apparently uncomplicated procedures^[^18^]^. While ultrasound improves safety and reduces carotid puncture rates^[^19^]^, cases of HS under real-time imaging have been documented^[^6,14^]^.

Diagnosis is primarily clinical. Pharmacological confirmation can be obtained by instilling one drop of 0.5% apraclonidine in each eye, with pupillary assessment at 30–45 minutes—reversal of anisocoria is diagnostic^[^20^]^. However, an explicit warning must be highlighted regarding apraclonidine use in very young infants under 6 months of age, as it can be absorbed systemically and cause severe adverse effects such as extreme somnolence, bradycardia, hypotension, and respiratory depression^[^21^]^. Given that the patient we report was only 38 days old, pharmacological testing with apraclonidine was avoided, and diagnosis relied strictly on clinical assessment. Alternatively, administration of 1–2 drops of 10% cocaine in each eye, with measurement at 50–60 minutes showing anisocoria ≥1.0 mm, confirms the diagnosis^[^22^]^. Imaging is indicated when the etiology is uncertain, to exclude neuroblastoma or carotid dissection^[^16^]^. In our case, alternative diagnoses including compressive hematoma, carotid artery injury, venous congestion, congenital anisocoria, intracranial pathology, and neuroblastoma-related HS were considered unlikely because of the abrupt postoperative onset, absence of local compressive signs, normal bedside ultrasonography, stable neurological examination, and rapid spontaneous recovery.

Prognosis is favorable, with most children recovering spontaneously within weeks to months^[^12,14^]^. However, some cases report residual anisocoria or mild ptosis^[^16,23,24^]^ (Table 1). In this infant, rapid improvement supported a transient neuropraxia rather than permanent injury.

Preventive strategies differ by route of access:

For percutaneous IJV cannulation, key measures include the following:

- Real-time ultrasound guidance^[^19^]^
- Limit head rotation to less than 30° to reduce IJV–carotid overlap^[^12^]^
- Avoid steep or medial needle angulation^[^10^]^ and minimize puncture attempts^[^25^]^.

For open surgical cutdown, as in the present case, the focus shifts to:

- Perform gentle dissection with minimal traction on the carotid sheath to avoid neuropraxia of the adjacent sympathetic chain^[^6,26^]^
- Avoid proximal venous ligation whenever possible, since ligation may cause ischemia or compression of the sympathetic fibers running within the perivascular adventitia^[^8,14^]^
- Meticulous hemostasis to prevent hematoma-related compression within the carotid sheath^[^9,10^]^
- Ensure close postoperative neurological monitoring, including early pupillary examination, to detect HS promptly^[^25,27^]^.

In both approaches, careful evaluation of the indication for IJV catheterization and a low threshold for alternative access sites (e.g., femoral vein) should be considered when cervical anatomy is unfavorable^[^23^]^.

## Conclusion

Iatrogenic HS following IJV catheterization in children is rare but clinically relevant. This case demonstrates that HS can occur even during open surgical placement under direct vision. Early recognition, reassurance, and management based on close observation and serial neurological and ophthalmological examinations typically result in full recovery. Increased awareness and preventive strategies are crucial for minimizing risk.

## Data Availability

Not applicable.
